# Estimating direct costs of the treatment for mucosal leishmaniasis in Brazil

**DOI:** 10.1590/0037-8682-0454-2020

**Published:** 2021-01-29

**Authors:** Janaína de Pina Carvalho, Tália Machado de Assis, Taynãna César Simões, Gláucia Cota

**Affiliations:** 1 Fundação Oswaldo Cruz, Instituto René Rachou, Grupo de Pesquisa Clínica e Políticas Públicas em Doenças Infecciosas e Parasitárias, Belo Horizonte, MG, Brasil.; 2 Centro Federal de Educação Tecnológica de Minas Gerais, Campus Contagem, Contagem, MG, Brasil.

**Keywords:** Mucocutaneous leishmaniasis, Cost analysis, Drug therapy, Meglumine antimoniate, Liposomal amphotericin B, Miltefosine

## Abstract

**INTRODUCTION:**

The objective of this study was to estimate the direct medical costs of the treatment for mucosal leishmaniasis (ML) using three therapeutic approaches in the Brazilian context.

**METHODS:**

We performed this economic assessment from the perspective of the Brazilian public healthcare system. The following therapeutic approaches were evaluated: meglumine antimoniate, liposomal amphotericin B, and miltefosine. Direct medical costs were estimated considering four treatment components: a) drug, b) combined medical products, c) procedures, and d) complementary tests.

**RESULTS:**

Treatment with meglumine antimoniate had the lowest average cost per patient (US$ 167.66), followed by miltefosine (US$ 259.92) in the outpatient treatment regimen. The average cost of treatment with liposomal amphotericin B was US$ 715.35 both in inpatient regimen. In all estimates, the drugs accounted for more than 60% of the total cost for each treatment approach.

**CONCLUSIONS:**

These results demonstrate the marked differences in costs between the therapeutic alternatives for ML. In addition to efficacy rates and costs related to adverse events, our data have the potential to support a complete cost-effectiveness study in the future. Complete analyses comparing costs and benefits for interventions will assist health managers in choosing drugs for ML treatment in Brazil as well as in establishing effective public health policies.

## INTRODUCTION

Mucosal leishmaniasis (ML) is a severe form of tegumentary leishmaniasis (TL) and, generally, a late complication of the *Leishmania (Viannia) braziliensis* infection that usually occurs years after the cutaneous form^1^ ^-^ ^3^. During 2016-2018, ML accounted for 4.22% (1,942 out of 44,383) of TL cases reported in the Americas, where 6 countries-Brazil, Colombia, Peru, Nicaragua, Bolivia, and Venezuela-accounted for 84% of TL cases^4^.

Several factors, such as the infecting *Leishmania* species and the host immune response, determine the differences in the extent and severity of the disease. The clinical manifestations of ML range from involvement limited to the nasal cavity to facial disfigurement and lesions in the oral cavity and lower airways; the latter can culminate in airway obstruction, aspiration, and death. From a social perspective, ML is associated with economic losses, stigmatization, and psychological problems^2^ ^,^ ^5^. 

One factor that can increase morbidity is the small therapeutic arsenal available for ML, which is administered exclusively parenterally and has high toxicity^2^ ^,^ ^3^ ^,^ ^6^. In Brazil, the Ministry of Health (MH) recommends the following drugs: meglumine antimoniate, liposomal and deoxycholate amphotericin B, and pentamidine^3^. More recently, the Pan American Health Organization included miltefosine to its list of strategic drugs; it is an oral drug with great ease of administration. Therefore, the National Committee for Health Technology Incorporation (CONITEC) of the MH recommended this drug in the Unified Health System (SUS) as one of the first-line treatment for TL, leading to its subsequent acquisition and adoption in 2021 by the Brazilian government^7^. The CONITEC evaluates the efficacy, safety, and cost-effectiveness before the incorporation of the new health technology in Brazil^8^ ^,^ ^9^. This process is also validated by the participation of the society and members of the scientific academy through public consultation^10^. These assessments help health managers to implement a transparent decision-making process, establish priorities, and allocate resources efficiently. These are important in the treatment of neglected tropical diseases (NTDs), such as ML because they mainly affect the poor and vulnerable populations in developing countries^11^ ^,^ ^12^. Despite this, there are limited economic studies addressing NTDs or, specifically, leishmaniasis^7^ ^,^ ^13^ ^-^ ^17^. Even if SUS adopts miltefosine in Brazil for TL, we currently lack the evidence demonstrating its efficacy, specifically for treating ML. 

Currently, a randomized, multicenter trial is underway in Brazil comparing miltefosine with liposomal amphotericin B, administered on consecutive days or on an intermittent schedule (Brazilian Clinical Trials Registry: RBR-5r93wn)^18^. The efficacy and safety data from this study have the potential to support future analytical studies on cost-effectiveness for ML. With the available data, we aimed to estimate the direct medical costs of the therapeutic options for ML-meglumine antimoniate and liposomal amphotericin B-currently recommended as the first-line treatment in Brazil in addition to miltefosine. This would be the first step toward establishing a cost-effective treatment for ML in Brazil.

## METHODS

### Study design

This economic evaluation was performed from the perspective of the SUS, considering the period between the pretreatment clinical assessment and the evaluation 6 months after treatment. Our study population consisted of the average annual number of confirmed cases of ML in Brazil, from 2014 to 2018 in *Sistema de Informação de Agravos de Notificação* - SINAN^19^, the epidemiological surveillance database of Brazilian MH.

To compare the costs of each therapeutic approach, we estimated the annual direct medical costs of using meglumine antimoniate, liposomal amphotericin B, and miltefosine, considering that all cases of ML in the study population were treated with each of these approaches and excluding cases with contraindications for use (Table 1 **)**. In addition to the total annual cost, the direct medical costs per patient treated with these approaches were calculated. Costs incurred for the diagnosis of ML were not considered.

**TABLE 1:** Therapeutic regimen, group of eligible patients, and complementary drugs needed for each treatment under evaluation.

Drug	Eligible patients	Dose	Level of care for drug administration	Assumptions	Combined medical products
Meglumine antimoniate: 5 mL ampule (81 mg Sb^+5^/mL)	Up to 49 years, without comorbidities or clinical contraindications*	20 mg/kg/day (up to a maximum of 1,215 mg or 3 ampules) for 30 days	Outpatient	1-3 ampules/day/ patient	Pentoxifylline (only for patients over 12 years, 400 mg orally 3 times daily for 30 days)
Liposomal amphotericin B: 50 mg ampule	Older than 50 years or with comorbidities*	3-5 mg/kg/day (up to a total cumulative dose of 25-40 mg/kg) for ~10 days	Inpatient	Daily dose of 3 mg/kg/day	-
				Total cumulative dose of 30 mg/kg	
				Number of ampules rounded to the integer above the calculation per kg	
Miltefosine: 50 mg capsules	Older than 12 years	Body weight <45 kg: 100 mg/day for 28 days	At home with outpatient follow-up	Contraceptive method for women of childbearing age eligible for treatment (12 to 55 years)	Contraceptive method Effective method: medroxyprogesterone, 150 mg/mL (before and after treatment)
		Body weight >45 kg: 150 mg/day for 28 days			Barrier method: male condom (1 per day; total: 180 units)

*comorbidities: renal, heart, or liver failure or others that compromise immunity; kidney, heart, or liver transplant; failure of treatment with meglumine antimoniate; and current pregnancy. **Sb** ^+5^ **:** pentavalent antimoniate.

### Cost estimates, assumptions, and data source

To calculate the costs related to each drug, we estimate their average doses per age group in both sexes based on therapeutic recommendations per kilogram of weight and average weight estimates (weighted by sex and age) of the Brazilian population. Furthermore, we considered the costs for accessory medical products recommended in combination with the drug. Table 1 summarizes the therapeutic regimens recommended for each approach and their specificities.

Four treatment components were included: a) drug, b) combined medical products, c) procedures, and d) complementary tests. Table 2 describes the therapeutic approaches, unit cost, and value of each component of each therapeutic approach. The average total cost per approach was calculated as the sum of costs of all components in all strata of eligible patients divided by the mean annual number of patients for that therapeutic approach.

**TABLE 2:** Description, unit cost, and quantity of the components considered for each therapeutic alternative studied.

Component	Description	Unit cost (US$)	Source*	Number per approach		
				Meglumine antimoniate	Liposomal amphotericin B	Miltefosine
	Number of patients considered	-	1	600	1075	1011
Drug	Meglumine antimoniate	1.23	2	**	**	**
	Liposomal amphotericin B	15.40	2	**	**	**
	Miltefosine	2.71	2	**	**	**
**Combined medical products**	Pentoxifylline	0.12	2	**	**	**
	Effective contraceptive*	2.74	3	**	**	**
	Barrier contraceptive*	0.06	3	**	**	**
**Procedure**	Medical visit in specialized care	2.53	4	6	2	6
	Administration of drugs in specialized care	0.16	4	30	-	-
	Treatment of other diseases due to protozoa - Hospitalization	35.17	4	-	1	-
	Daily cost of stay over the standard length of hospitalization	5.08	4	-	5	-
**Complementary tests**	**Cardiac monitoring:** Electrocardiogram	1.31	4	5	-	-
	**Hematopoietic function:** Hemogram	1.04	4	5	1	-
	**Renal function:** Urea	0.47	4	5	1	6
	Creatinine	0.47	4	5	1	6
	**Liver function:** Oxaloacetic transaminase	0.51	4	5	1	5
	Pyruvic transaminase	0.51	4	5	1	5
	Total bilirubin and fractions	0.51	4	5	1	5
	Alkaline phosphatase	0.51	4	5	1	-
	**Pancreatic function:** Amylase	0.57	4	5	-	-
	Lipase	0.57	4	5	-	-
	**Serum electrolytes:** Potassium	0.47	4	-	1	-
	Magnesium	0.51	4	-	1	-
	Beta-HCG***	1.99	4	1	1	7

**US$:** United States dollar; **HCG:** serum human chorionic gonadotropin. *Source: 1 = National Notifiable Diseases Information System; 2 = Ministry of Health; 3 = Data Bank of Hospital Prices; 4 = Management System of the Table of Procedures, Medications, and Orthoses, Prostheses and Special Materials. **Variable, by age, weight, and sex. ***For women of childbearing age (10 to 55 years).

We employed a top-down cost estimation approach that uses aggregated data of global costs for the set of treated cases available in the MH databases and market price records^20^. All costs were obtained for 2019 as the base year in Brazilian reais (R$) and converted to US dollars (US$), using the 2019 annual average commercial exchange rate for sale (R$ 3.9451 = US$ 1.00)^21^. The costs in other years were adjusted based on the official inflation rate determined by the cumulative Extended National Consumer Price Index^22^.

We considered costs of pharmaceutical units of each of the three drugs as reported by the General Coordination of Pharmaceutical Assistance and Strategic Drugs of the Department of Pharmaceutical Assistance and Strategic Inputs of the MH. The costs of contraception methods were obtained from the *Banco de Preços em Saúde* - BPS, Brazilian database of the MH^23^, and those of procedures (medical visit, hospitalization, drug administration) and complementary tests from another SUS database, named *Sistema de Gerenciamento da Tabela de Procedimentos, Medicamentos e Órteses, Próteses e Materiais especiais* - SIGTAP^24^ ^,^ ^25^.

The therapeutic details (recommended dose, specific contraindications, and need for a combined product) of each approach were established according to the recommendations of the MH or, when absent, according to the manufacturers’ recommendations^3^ ^,^ ^26^ ^-^ ^30^. The dose for each approach depended on the weight of patient. Therefore, for an accurate estimation, we calculated the total cost of each treatment as the sum of costs of the subgroups of ML patients, stratified by age group and sex, for which weighted-average body weights were estimated based on data from the Brazilian Institute of Geography and Statistics^31^ ^-^ ^33^ (Table 3 and Supplementary Material). We described additional definitions needed for cases with unavailable information and those with more than one recommended approach, as follows: 

**TABLE 3:** Annual average number of patients with mucosal leishmaniasis in Brazil and average body weights by age group and sex from 2014 to 2018.

Age group (years)	Annual average	Annual average	Annual average	Average male	Average female	Sex-weighted
	number of ML	number of ML	number of ML	body weight	body weight	average body
	cases (males)	cases (females)	cases (Total)*	(kg)	(kg)	weight (kg)
<1	9	3	11	8.1	7.5	8.0
1-4	6	7	13	14.9	14.4	14.6
5-9	14	11	24	25.3	25.2	25.3
10	4	2	6	33.4	34.3	33.7
11	6	4	10	36.8	39.5	37.9
12	3	2	6	42.0	44.2	42.9
13	5	2	7	47.4	47.9	47.5
14	9	2	11	52.3	50.0	51.8
15-19	36	11	47	62.3	54.3	60.4
20-39	228	63	291	76.4	66.1	74.2
40-44	64	16	81	77.8	69.3	76.1
45-49	72	21	93	77.2	68.6	75.3
50-54	73	21	94	76.4	68.6	74.7
55-59	64	19	82	75.5	68.9	74.0
60-64	68	15	83	74.8	67.0	73.4
65-69	56	16	72	73.1	66.3	71.6
70-79	72	26	98	70.2	63.2	68.3
≥80	33	13	46	66.6	58.9	64.4
Total	822	254	1075	-	-	-

**ML:** mucosal leishmaniasis. *Some sums do not match due to rounding annual case averages.

### A. Meglumine antimoniate

1. The Brazilian MH recommends this drug for patients up to 49 years of age, combined with pentoxifylline. The latter was approved only for patients over 12 years of age^3^ ^,^ ^28^ ^,^ ^29^.
2. An outpatient treatment regimen was assumed in this study, considering that ML does not require hospitalization.
3. Certain contraindications were considered for some comorbidities (renal, cardiac, hepatic, or other impairments of immunity) and specific clinical conditions (kidney, heart, or liver transplant; therapeutic failure of meglumine antimoniate; and current pregnancy)^3^ ^,^ ^29^ (Table 1). In these cases, MH recommends liposomal amphotericin B as the first choice of drug. To address these contraindications, we consulted with MH for information on the release of liposomal amphotericin B in patients with ML up to 49 years of age. The data indicated a release rate of 5.5% in this age group - 33 releases in 600 cases of ML, which did not affect the final calculations of the present study^34^.

### B. Liposomal amphotericin B

1. We considered all patients with ML as eligible for liposomal amphotericin B treatment because no associated condition or comorbidity is an absolute contraindication to its use^3^ ^,^ ^30^.
2. We assumed that this approach required hospitalization because liposomal amphotericin B is exclusively administered intravenously in a hospital setting, and ML is not eligible for outpatient treatment under the SUS.
3. The total cost of hospital stay for amphotericin B-related ML treatment was calculated as the sum of the value provided in the SUS refund table under “hospitalization for treatment of diseases by protozoa” that corresponds to a 5 day-stay, and five more daily rates and multiplied by the number of ML patients eligible for this treatment.
4. A dose of 30 mg/kg was defined as the standard as the recommended dose in Brazil ranges 25-40 mg/kg^3^. This dose is usually used in clinical practice^35^ and requires an average of 10 days of hospitalization.

### C. Miltefosine

1. It is recommended only for patients older than 12 years of age because there is no data on its efficacy or safety in pediatric populations. There are no restrictions on its use, except in cases of allergic reactions to the drug, pregnancy, and Sjögren-Larsson syndrome^26^ ^,^ ^27^. As it can be orally administered, we assumed that the patient would self-administer it with medical monitoring on an outpatient basis. The dose recommended by the manufacturer and determined according to the patient’s weight range (below or above 45 kg) was used as the parameter.
2. Miltefosine is potentially teratogenic, and its inadvertent use is prohibited in women of childbearing age (younger than 55 years). Thus, the possibility of pregnancy should be excluded before treatment, and at least two contraceptive methods (one highly effective and one barrier method, as required by the MH) should be used for 30 days before the start of treatment, throughout the treatment, and 4 months after the end of treatment^26^, for a minimum of 180 days of contraception. Given the diversity of available contraceptive methods, we considered medroxyprogesterone acetate (150 mg/mL) injection as the highly effective method for quarterly use due to its high efficacy (prolonged action and supervised adherence), assuming two applications. As the barrier method, we chose the male condom as it is the most widespread method in Brazil and estimated a supply of one daily unit for a total of 180 days^36^.

### D. Complementary tests

1. We defined the necessary complementary tests before and during each type of treatment based on the current recommendation of the MH and the official recommendations of the respective manufacturers. In the case of divergent recommendations, the most specific or conservative recommendation was adopted. To define the periodicity of laboratory monitoring when not clearly specified, we chose a weekly interval from the beginning of treatment.
2. The laboratory component of treatment with liposomal amphotericin B was counted separately in pretreatment, as the hospitalization package already included procedures for drug administration and professional evaluation.
3. In addition to the hematological, biochemical, and electrocardiographic tests, all women of childbearing age (10-55 years) had to undergo a high-sensitivity pregnancy test (serum human chorionic gonadotropin - beta-HCG - measurement) before starting treatment, regardless of the drug to be used. For miltefosine, we followed the recommendation of the National Health Surveillance Agency (Anvisa)^26^; in addition to testing 30 days before the start of treatment, pregnancy testing was consider to be repeated periodically during and 4 months after the end of treatment, resulting in a minimum of 7 beta-HCG measurements.

### Sensitivity analysis

To assess the impact of variations in model parameters on the results and to estimate the reliability of these results^37^, we varied drug costs and subgroups of patients eligible to receive treatment with liposomal amphotericin B. Thus, we performed a sensitivity analysis assuming an arbitrary variation of 25% in costs and changing the selection criteria of the population eligible for this treatment. Regarding the latter, its annual cost was calculated only for patients with contraindications to meglumine antimoniate, i.e., age > 49 years, which represented a mean eligible population of 475 patients with ML per year.

## RESULTS

Table 4 lists the direct medical costs for each therapeutic approach. We estimated the total annual costs of treatment for all patients eligible for meglumine antimoniate, miltefosine, and liposomal amphotericin B at US$ 100,607.68, US$ 262,826.52, and US$ 769,341.17, respectively. The average cost of the treatment per patient was US$ 167.66, US$ 259.92, and US$ 715.35, respectively. In all estimates, the drug component accounted for more than 60% of the total cost for each therapeutic approach: 63%, 87.5%, and 90% for meglumine antimoniate, miltefosine, and liposomal amphotericin B, respectively.

**TABLE 4:** Direct medical costs of the three therapeutic approaches for the treatment of patients diagnosed with mucosal leishmaniasis in Brazil.

Component	Meglumine antimoniate			Liposomal amphotericin B			Miltefosine		
	Average total	Average cost/patien	%	Average total	Average cost	%	Average total	Average cost	%
	cost/year	t		cost / year	/patient		cost /year	/patient	
	(US$)	(US$)		(US$)	(US$)		(US$)	(US$)	
**Drug**	63,411.22	105.67	63	693,063.50	644.43	90	229,850.15	227.30	87.5
**Combined medical products**	5,553.22	9.25*	5			0	759.16**	0,75**	0.3
							1,492.16***	1.48***	0.6
**Procedure**	12,001.14	20.00	12	70,616.80	65.66	9	15,379.08	15.21	5.9
**Complementary tests**	19,642.10	32.73	20	5,660.86	5.26	1	15,345.97	15.18	58
**Total**	**100,607.68**	**167.66**	**100**	**769,341.17**	**715.35**	**100**	**262,826.52**	**259.92**	**100**
**(Variation 25%)**	**(75,455.76-125,759.61)**	**(125.74-209.57)**		**(577,005.87-961,676.46)**	**(536.51-894.19)**		**(197,119.89-328,533.15)**	**(194.94-324.89)**	

**US$:** United States dollar. *pentoxifylline; **medroxyprogesterone; ***male condom.

The 25% variation in the annual costs of drugs for the treatment of all eligible cases demonstrated that these ranged from US$ 75,455.76 (for meglumine antimoniate) to US$ 961,676.46 (for liposomal amphotericin B). The average cost of treatment per case ranged from US$ 125.74 (for meglumine antimoniate) to US$ 894.19 (for liposomal amphotericin B). Considering only patients with contraindications to meglumine antimoniate as eligible for liposomal amphotericin B treatment, the average total cost of the treatment was US$ 349,010.89/year, with an average of US$ 734.14 per patient. These are only 3% higher than the average costs considering the entire population with ML (US$ 715.35 per patient) and are within the 25% variation range (US$ 536.51-894.19).

## DISCUSSION

ML poses a serious public health problem, and its progressive and destructive nature leads to extensive morbidity^3^ ^,^ ^38^. We lack adequate research in ML and public policies aimed at coping with it are scarce, despite the extensive characterization of its insufficient therapeutic spectrum^3^. Similar to the trials addressing efficacy and safety, there are limited partial (cost estimation) or total (cost-effectiveness analysis) economic studies focusing on ML.

Cost estimation is one of the first steps in economic assessment that calculates the costs involved in a given health intervention^39^. In this study, we evaluated the direct medical costs of the therapies currently available in Brazil for ML and an oral alternative in the phase of incorporation in Brazil (a phase III study is in progress), although not yet officially recommended for ML. 

We calculated the mean cost of treatment for each of the three drugs and the total annual costs of these treatments, for all eligible patients, from the perspective of SUS. Meglumine antimoniate requires the lowest investment per patient (US$ 167.66), followed by miltefosine (US$ 259.92), and liposomal amphotericin B (US$ 715.35), the latter being the most expensive of the three. For each of these approaches, the cost of drug accounted for the major expense. Additionally, factors that increased the medical costs of a treatment are the need for hospitalization for drug administration and specialized professionals, requirement for laboratory monitoring, directly related to its frequency and complexity and the rate and severity of adverse events that may require prolonged hospital stay and additional costs. 

Our results are partially consistent with those of Mistro et al. (2017), who conducted an economic analysis in a hospital in northeastern Brazil^40^. They reported that the cost of successfully treating ML with pentavalent antimonial drugs was US$ 1,154.92, lower than for liposomal amphotericin B (US$ 10,265.37). For liposomal amphotericin B, the cost of drug (US$ 9,711.51) formed the majority expense. In contrast, the cost of hospitalization (US$ 1,060.98)^40^ was the primary cost in pentavalent antimonial treatment, which we did not consider in this study as we modeled its administration on an outpatient basis.

This marked differences in costs of different therapies reinforces the need to determine direct medical costs, costs due to adverse events, and their efficacies in making therapeutic choices, especially in the context of public health policies. Despite the high cost, liposomal amphotericin B presents a safety profile more favorable than that of meglumine antimoniate and deoxycholate amphotericin B, thus being a unique option for some patients with comorbidities^41^. Therefore, we need to consider successful therapeutic rates and avoided deaths in a comprehensive analysis. Contrararily, costs must be critically analyzed from a global perspective. Liposomal amphotericin B acquisition is currently subsidized by the World Health Organization^42^, especially in the endemic and undeveloped countries. Treatment with this drug displayed the highest estimated direct cost in both our study and that by Mistro et al. (2017). These findings highlight the dependence on and the monopoly of a single drug manufacturer, which can affect both cost and access to treatment^14^. The current context of ML treatment, based on drug production and distribution logic marked by strong international industry dependency and small margin for cost reduction, makes the poorest leishmaniasis endemic countries even more vulnerable. Considering the difficulty in identifying new drugs against *Leishmania,* the transfer of technologies and the incentive for development of a national industry have emerged as promising initiatives, given the opportunity offered by the expiration of the patent in 2016 of Ambisome^®^ produced by Gilead, the main manufacturer of liposomal amphotericin B worldwide^43^.

The second and more relevant component of the cost of ML treatment was the cost of complementary tests for monitoring treatment toxicity, representing 20% and 6% of the average cost for the meglumine antimoniate and miltefosine, respectively. For liposomal amphotericin B, we could not determine this cost because it was included in the hospitalization package for the treatment for ML, according to the SUS guidelines, corresponding to 9% of the total cost of treatment.

Our analysis confirmed that the additional medical products recommended in combination with meglumine antimoniate (i.e., pentoxifylline) and miltefosine (i.e., contraception) added marginal cost to the treatments with 5% and less than 1%, respectively. We did not account for the contraception costs in female patients treated with antimony or liposomal amphotericin B, as this is not a formal recommendation of the manufacturers or the Brazilian treatment guide. However, to avoid drug use during the first trimester of pregnancy, we recommended contraception regardless of the option chosen for ML treatment. Despite being included for all therapeutic approaches, due to its low cost, it would not affect the results. Notably, the cost of contraceptive methods chosen by women can vary widely and will not necessarily correspond to this study model.

Although laboratory monitoring with complementary tests corresponded to a maximum of 20% of the treatment cost, this study demonstrated significant differences in their economic burden, which was at least twice as high for meglumine antimoniate as for the two other therapeutic approaches. Due to lack of data, we could not consider treatment costs of eventual adverse events for each approach. The occurrence of adverse events will likely require complementary tests at a shorter time interval for their monitoring and management, further increasing the costs involved or resulting in early treatment interruption. Additionally, the cost of treating complications arising from the three types of therapeutic approaches would be different.

The complexity of the health system was our main assumption in defining the types of procedures required for each therapeutic approach. By defining the administration of meglumine antimoniate as an outpatient procedure in all cases, we expect some underestimation of costs, as cases that would require hospitalization are not represented. Assuming treatment with meglumine antimoniate in the inpatient regimen for 30 days, the estimated cost is US$ 167.36, which is about thrice the sum of the costs in the outpatient regimen involving medical visits and drug administration (US$ 20.00) and complementary tests (US$ 32.73).

In contrast, the adoption of inpatient treatment for liposomal amphotericin B may have overestimated the number of hospitalizations, since it is also possible to administer it on a day-hospital basis. Inversely, the estimated daily hospital stay required for ML treatment (10 days) may have been lower than that actually required, as we did not account for the need for temporary interruption of procedure due to tests for kidney function, a frequent event during the treatment of elderly patients. Mistro et al. (2017) analyzed actual lengths of hospital stay^40^, where the average length for treatment with liposomal amphotericin B was 56.8 days.

The MH does has not yet recommend miltefosine as a treatment for ML; therefore, the definitions adopted in this study may not correspond to its actual use in Brazil. In the future, if approved for TL treatment, an important concern will be the risk of miltefosine resistance, as has been demonstrated for visceral leishmaniasis, considering its easy access and less control on use^44^.

Some aspects make this assessment useful, especially in the Brazilian context, such as top-down cost estimation using aggregated data provided by SUS. It allows comparisons between different studies at the regional or national level^20^ and assists in decision-making over a wider scope. However, amounts paid in the public health system are usually much lower than the market values, limiting the extrapolation of these conclusions to other scenarios, such private perspective or other countries. Decisions related to ML treatment on an outpatient basis is another crucial point that influences cost in the same way as the toxicity monitoring and clinical follow-up protocols adopted in each region. Most importantly, direct medical costs account for only a fraction of the expenses involved in ML therapy; the total expense would include direct costs paid by the society (e.g., travel expenses and work days lost) and the costs due to adverse events to the drugs. 

This study presents unpublished data on direct costs of therapeutic approaches for ML available in the Brazilian public healthcare system. These results highlighted the marked cost differences between the different therapeutic alternatives for ML, emphasizing the need for studying their cost-effectiveness. Additionally, a detailed analysis of all cost-generating components in a therapeutic approach can identify possible aspects that can be reduced, modified, or made cheaper. In the future, complete analyses comparing costs and benefits for interventions will assist health managers in the process of choosing drugs for ML treatment in Brazil as well as in establishing effective public health policies for disease management. More economic studies focusing on ML treatment are ongoing and will complement the scientific evidence generated in our study.
