# Supplementary material for: Estimating direct costs of the treatment for mucosal leishmaniasis in Brazil
Source: Rev Soc Bras Med Trop. 2021 Jan 29;54:e04542020. doi: 10.1590/0037-8682-0454-2020 (PMC7849328; doi:10.1590/0037-8682-0454-2020)
Supplement: Supplementary file 1 [file 1678-9849-rsbmt-54-e04542020-suppl1.pdf]

Supplemental File: Estimating direct costs of the treatment for mucosal leishmaniasis in Brazil

Estimated average weight of patients with mucosal leishmaniasis

The weight of patients with mucosal leishmaniasis (ML) is not included in the aggregated information in the National Notifiable Diseases Information System (SINAN)[19]. Therefore, we obtained data from the last survey by the Brazilian Institute of Geography and Statistics (IBGE). Specifically, we used the table of population estimates of the median height and weight of children, adolescents, and adults, by sex, position in the family, and age in Brazil and Great Regions (Table 2645) produced by the 2008–2009 Family Budget Survey[31]. However, the age groups in SINAN data to present the age distribution of ML are different from those in the IBGE data. Thus, we made the following approximations: a) body weights of individuals below 20 years of age were calculated as the mean of the medians of the weights reported for each age year, as provided by the IBGE and b) body weights for individuals above 20 years were calculated based on the 2013 National Health Survey, considering sample weights and the design effect due to the complex sampling plan of that survey, using the *survey* package of the statistical software R[32,33]. Table 3 displays body weights of patients with ML in the period from 2013 to 2017 by age group and weighted by sex. The sex-weighted average body weight was used to calculate the dose of each drug.
